# Swimming as Treatment for Osteoporosis: A Systematic Review and Meta-analysis

**DOI:** 10.1155/2020/6210201

**Published:** 2020-05-15

**Authors:** Yanlin Su, Zhe Chen, Wei Xie

**Affiliations:** ^1^Department of Emergency Medicine, Shandong First Medical University, No. 366, Taishan Street, Taian, Shandong 271000, China; ^2^South China Normal University, No. 55, Zhongshan Avenue, Guangzhou, Guangdong 510631, China

## Abstract

Osteoporosis is a chronic disease that seriously affects human health and quality of life. This study is aimed at determining whether swimming had an effect on the bone mineral density (BMD) of the spine and femoral neck in postmenopausal and premenopausal osteoporosis patients. We retrieved relevant literature and analyzed data from randomized controlled trials to assess the effect of swimming on BMD in postmenopausal and premenopausal women. Relevant studies, with no language restrictions, from inception to September 2019, were retrieved from the PubMed, Cochrane, EMBASE, and EBSCO databases independently by two investigators. The keywords used for the literature search were “osteoporosis” and “swimming.” The main results included BMD and *T*-score. We searched 256 relevant articles and finally screened five articles, including 263 participants. Lumbar spine density was mentioned in three articles. Although the heterogeneity of lumbar vertebral density is moderate, the analysis of swimmers to nonswimmers shows that the lumbar vertebral density in swimmers is improved [heterogeneity: chi^2^ = 5.16, df = 2 (*P* = 0.08); *I*^2^ = 61%]. We analyzed the following heterogeneous subgroups: subgroup 1 (3–6 hours) and subgroup 2 (<3 hours). The BMD in subgroup 1 was significantly higher than that in the placebo, while no effect on BMD was found in subgroup 2 [heterogeneity: chi^2^ = 0.15, df = 3 (*P* = 0.70); *I*^2^ = 0%]. According to the current evidence, swimming may improve the BMD of postmenopausal women participants, if the swimming time is between 3 and 6 hours, especially in long-term swimmers. However, the effectiveness of swimming does require further investigation.

## 1. Introduction

Osteoporosis is a musculoskeletal disease characterized by decreased bone mass and destruction of bone microstructure. Osteoporosis has many causes, including age, genetic factors, hormone therapy, and long-term bed rest. From an epidemiological point of view, osteoporosis mainly occurs in postmenopausal and premenopausal women and older men aged >50 years. Osteoporotic fracture is extremely harmful to older people; a hip fracture in older people is called the “last fracture” in life. In addition, 30% of women and 20% of men aged >50 years experience fractures [1]. With advancing age, human glands change to varying degrees, resulting in changes in hormone secretion [2]. Such changes lead to the breakdown of the original balance in the body and cause dysfunction of various organs, including bones. This disorder is manifested in the skeleton, mainly due to the increase or decrease in osteoclast and osteoblast activities, resulting in a decrease in bone mineral density (BMD). Moreover, the effect of decreased mobility on the skeleton should not be underestimated. For example, decreased mobility can lead to skeletal muscle atrophy, which increases the risk of fracture. At present, drug treatment for osteoporosis has been improved; i.e., monoclonal antibodies are used to block signal molecules of osteoblasts or osteoclasts to promote their role [3]. Clinical trials have confirmed its role and proved that it provided favorable effects on osteoporotic fracture. Sequelae of long-term drug use such as mandibular necrosis occur occasionally [4].

Meanwhile, studies reported that exercise intervention in osteoporosis may be a better treatment option [5]. Exercise therapy for osteoporosis aims to enhance the bone's ability to bear a considerable degree of load and tension [6], and it includes weight lifting, plyometrics, or other high-impact activity [5]. People of different age groups should be given individualized treatment; for example, in osteoporosis patients, appropriate load-bearing and tension should be employed to prevent continuous loss of bone mass and secondary injury, such as fracture [7]. At the same time, osteoporosis is usually accompanied by cardiovascular and cerebrovascular diseases, and intensive exercise is not suitable for these patients. Theoretically, although bone stimulation within a certain range is positively correlated with exercise intensity, bone stimulation can promote osteogenesis and increase bone mass [8].

However, for osteoporosis patients, the responsiveness of all organs, including bone, to external stimuli is lower than that of young people [9]. As an exercise therapy, swimming is expected to become a suitable physical activity to prevent bone loss in osteoporosis patients, although current studies have shown that swimming has no significant effect on improving bone mass in these patients.

At present, sports can be roughly divided into weight-bearing and non-weight-bearing sports. The National Osteoporosis Foundation of the United States recommended that high- and low-intensity weight-bearing training should be carried out at the same time for skeletal load, at least 30 min a day for 5–7 days a week. Moreover, attention should be paid to the muscle target of the exercise. Strong muscles can intensify the auxiliary role of the bones. It can improve posture, reduce falls, and promote bone metabolism [6].

Swimming, as a sport suitable for all ages, is rapidly becoming accepted by the general population. Studies have shown that swimming can improve cardiopulmonary function, reduce blood lipid levels, and improve body's antioxidant capacity, as well as delay aging. Previous studies have shown that swimming is a non-weight-bearing exercise and has no effect on bone mass. However, Orwoll et al. suggested that long-term adherence to swimming is beneficial to increase bone mass in older people [10] and the BMD of older men who are 3 years older than the male control group. With this, whether swimming can be used as a treatment for osteoporosis is controversial. In addition, there are some different opinions about swimming for the treatment of paralysis.

While some hypothesize that swimming has an effect on the bone density of patients with osteoporosis, others think it has no effect. Thus, a meta-analysis is needed to summarize past clinical studies on swimming and osteoporosis. This study is aimed at determining whether swimming has an effect on the BMD of the spine and femoral neck in postmenopausal and premenopausal osteoporosis patients.

## 2. Materials and Methods

### 2.1. Search Strategy

This meta-analysis was based on the PRISMA statement. Relevant studies, with no language restrictions, from inception to September 2019, were retrieved from the PubMed, Cochrane, EMBASE, and EBSCO databases independently by two investigators. The keywords used for the literature search were “osteoporosis” and “swimming.” The inclusion criteria were as follows: (1) clinical trials involving comparison of swimmers with inactive subjects and (2) BMD data being provided. The exclusion criteria were as follows: (1) clinical trials without control group, (2) osteoporosis was due to causes other than postmenopausal and premenopausal osteoporosis, and (3) animal studies of osteoporosis.

In this study, the quality of randomized controlled trials was assessed by two independent researchers using the Cochrane risk-of-bias tool. Quality indicators are divided into low risk, high risk, and unclear risk. The features of interest of the Cochrane manual include sequence generation, allocation sequence concealment, blinding, incomplete outcome data, selective outcome reporting, and other sources of bias. Articles are classified as high quality, medium quality, and low quality according to the following criteria: (1) If randomized sequence generation and allocation concealment are identified with high risk of bias, studies are graded as low quality. (2) When randomization and allocation concealment are considered to have low risk of bias, articles are rated as high quality. Evaluation of other features was excluded. (3) If these two criteria are not met, the literature will be rated as ambiguous.

### 2.2. Data Extraction

Two investigators (Y.S. and Z.C.) extracted data from the identified article, including the study title, journal, country, design, mean age, sample size, and relevant outcomes. If the selected articles contained two or one more groups of data, only relevant data were extracted for analysis. If there are differences between the two investigators, such differences were settled through consensus.

The primary indicators were bone density in the lumbar spine, upper extremity, lower extremity, and femoral neck.

### 2.3. Statistical Analysis

Extracted data were analyzed using RevMan 5.3.5 (Copenhagen: The Nordic Cochrane Centre, The Cochrane Collaboration, 2014). The mean difference (MD) and 95% confidence interval (CI) were used for continuous variables. Cochrane's *Q* and *I*^2^ were used to test the heterogeneity of our data. When *P* > 0.1 and *I*^2^ < 50%, the fixed effect model was used. When *I*^2^ > 50% and *P* < 0.1, the random effect model was used.

## 3. Results and Discussion

### 3.1. Results

**Figure 1:**
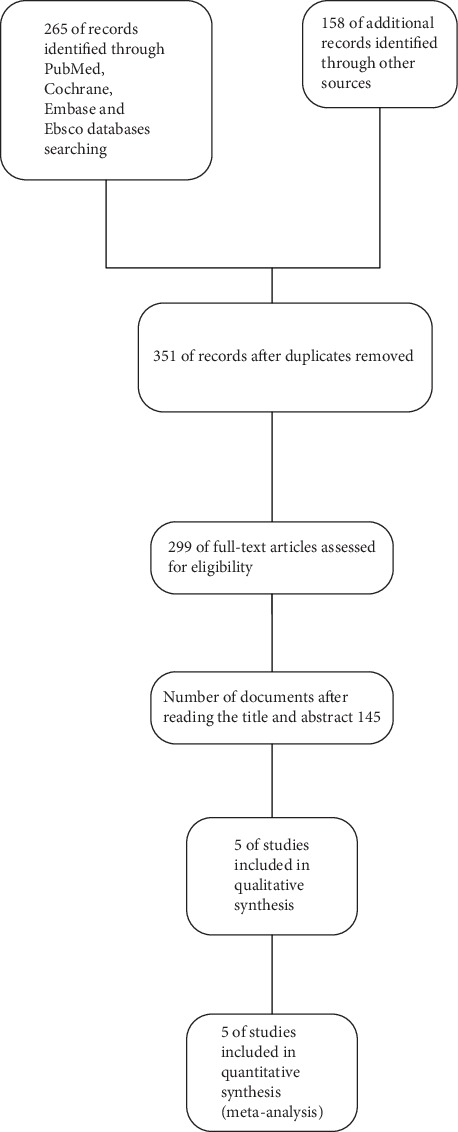
Flow diagram of the study selection process.

Our literature search retrieved a total of 423 articles. After removing duplicates, the remaining 351articles were examined. Article title, abstract, and full text were read; finally, five articles met the inclusion criteria, and the total number of participants was 263 (Figure 1).

We also summarized the basic information of the five articles and presented them in Table 1.

**Figure 2:**
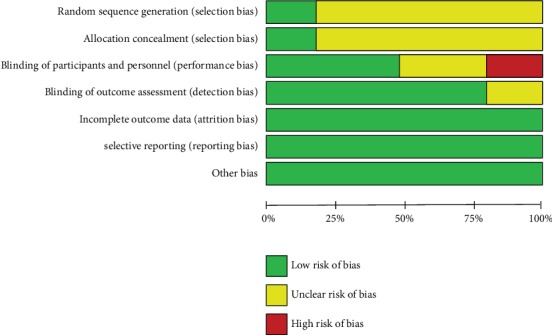
Assessment of risk of bias in all included randomized controlled trials.

**Figure 3:**
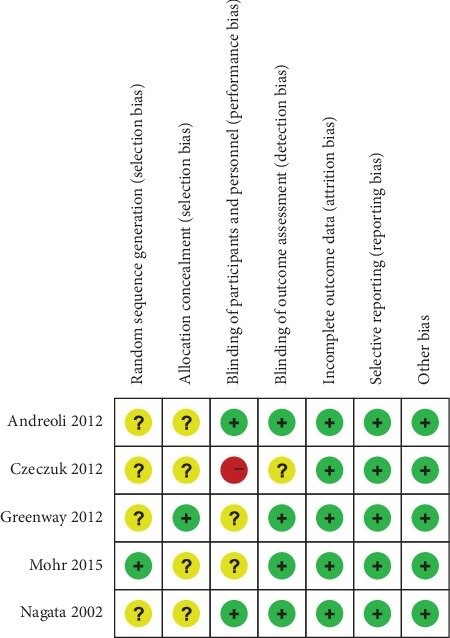
Assessment of risk of bias in all included randomized controlled trials.

The included articles were published between 2002 and 2015. All included participants were women aged >40 years. The risk assessments of all five articles are shown in Figures 2 and 3. Randomization was clearly reported in all randomized controlled experiments, but no article mentioned the randomization method. All trials were completed within the trial period.

**Figure 4:**
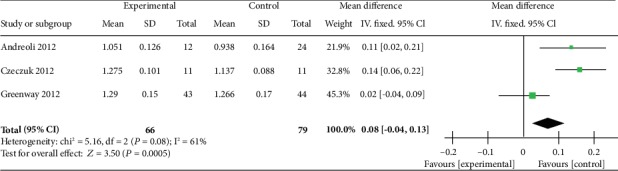
Forest plot of meta-analysis showing the effect of swimming on the bone mineral density of the lumbar spine.

**Figure 5:**
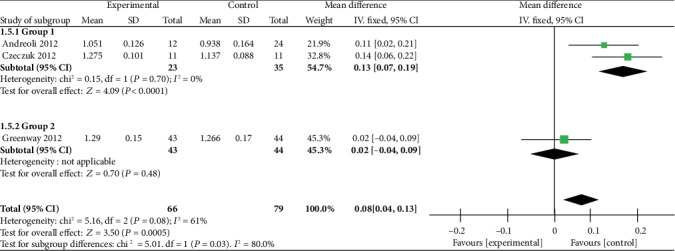
Forest plot of meta-analysis showing the effect of swimming on the bone mineral density of the subgroup of the lumbar spine.

Of the five studies, the three measured the BMD of the lumbar spine. After analyzing data of the three studies, although the overall lumbar spine density of the experimental group was significantly higher than that of the control group, we found that the data of the three studies showed medium heterogeneity [heterogeneity: chi^2^ = 5.16, df = 2 (*P* = 0.08); *I*^2^ = 61%] (Figure 4). Given the high heterogeneity of the lumbar spine density results, we performed subgroup analysis. We divided the participants with lumbar BMD into subgroup 1 and subgroup 2. Subgroup 1 was composed of postmenopausal women (*n* = 35) with swimming time of 3–6 h per week, while subgroup 2 was composed of premenopausal women (*n* = 44) with swimming time less than 3 h. Two articles included a postmenopausal swimmer group, and one article included a premenopausal swimmer group. We found that the lumbar spine density of postmenopausal swimmers in the experimental group was significantly higher than that in the control group [heterogeneity: chi^2^ = 0.15, df = 1 (*P* = 0.70); *I*^2^ = 0%], while in the general population, the trend was not significant (Figure 5).

## 4. Discussion

Our results suggest that swimming may have an effect on the BMD of postmenopausal swimmers if the swimming time is between 3 and 6 h, but not in premenopausal swimmers with swimming time less than 3 h. This may prove wrong the notion that swimming does not increase BMD in osteoporosis.

At present, many studies report on the effect of swimming on osteoporosis; most of which support that swimming does not improve BMD. However, some experiments have confirmed that it affects not only BMD but also the level of bone turnover markers, such as CTX (decreased bone resorption marker) [11].

Our results also suggest that swimming, as a fitness program, may have an effect on BMD. Although only one trial has reported biomarkers and no data can be compared, we believe that the effect of swimming on bone turnover markers cannot be underestimated. Thus, more clinical trials on the effects of swimming are needed.

We believe that the effect of swimming on osteoporosis is mainly reflected in the following aspects. First, swimming stimulates osteoblasts by inducing muscle movement and water pressure on the bone, which ultimately delay bone mass decline. Second, swimming may affect the balance of bone mass regulation by increasing the content of estrogen in the body. Studies have shown that the levels of testosterone and estradiol in the blood of swimming trainers are significantly higher than those of the control group [12]. In a certain range, the content of sex hormones is positively correlated with swimming time. Sex hormones can promote the formation of bone matrix, increase bone salt deposition, and ultimately increase bone mass [13]. Third, swimming can promote blood circulation throughout the body. Swimming can accelerate blood renewal in the bone cortex and keep the balance of blood in the bone. Such an environment is conducive to bone formation but not to osteolysis, promoting osteogenesis. Finally, swimming can increase gastrointestinal peristalsis, appetite of older people, and increase vitamin D formation, thereby increasing calcium absorption. Increased calcium in the blood inhibits release of calcium from the bone to blood and reduces bone loss [12].

Because there are differences in the experimental design among the three articles which report the data of lumbar BMD, we designed a subgroup analysis design in the experimental design, which is based on age (or menopause, i.e., premenopausal and postmenopausal groups) and exercise time (3–6 h in subgroup 1, <3 h in subgroup 2). It is not clinically reasonable to group participants according to age to explain the moderate heterogeneity, i.e., the increase of BMD decreases with age, because as we aged, the sensitivity of bones to forces decreases. It seems acceptable to explain the heterogeneity from the perspective of exercise time. Previous studies have also confirmed that bone growth and development are directly related to exercise time. Exercise can increase muscle contraction. In a proper range, as you increase the exercise time, muscle contraction will also be strengthened, so the effect of muscle on bone will also be enhanced. Exercise can also accelerate blood circulation, increase metabolic efficiency, and reduce the negative effects of obesity on bone. In proper time, increasing exercise time can enhance BMD [6].

Bone tissue is a hard connective tissue composed of bone cells, fibers, and matrix. A large amount of calcium salt is deposited in the mechanism, which can play a supporting role. Osteocytes responded significantly to external loading or mechanical loading. The protruding processes of the osteocytes are embedded in the bone matrix to support the load of the whole matrix [14].

The regulation of sex hormones on bone may be achieved by regulating the estrogen receptor of osteoblasts. In animal experiments, the osteoclast activity of castrated mice was increased [15].

Swimming may affect osteoporosis by means of low-intensity vibration. Under the no-load condition, low-intensity mechanical signals can promote bone formation [16], and low-intensity mechanical signals can inhibit the production of fat and reduce triglycerides levels in the blood [17]. Moreover, adipose cells and osteoblasts share a common progenitor cell and mesenchymal stem cells [18]. Low-intensity vibration has a certain effect on reversing adipocyte production and bone dissolution. At the same time, low-intensity vibration can reduce the effects of obesity on the human immune system. This low-intensity vibration is caused by muscle contraction on the skeleton [19]. In in vitro experiments, osteoblasts stimulate osteocalcin secretion and osteopontin increase under mechanical loading, thus promoting matrix mineralization. When stimulated by shear stress, the beta-catenin signal in osteoblasts was upregulated [15, 20].

Human experiments have confirmed that low-intensity vibration can promote bone mass in disabled children. It also promotes the synthesis of skeletal muscle and skeleton in osteoporotic women aged 15–20 years and can regulate bone balance in women with anorexia nervosa [21]. The Food and Drug Administration defines low-intensity vibration as a low-risk form of exercise that lasts up to 4 h. Therefore, low-intensity vibration is suitable for older patients or patients with spinal cord injury [21].

Swimming may ultimately reduce inflammatory bone loss by reducing the inflammatory state associated with obesity. Experiments have shown that obesity increases the number of osteoblasts and lymphocytes and decreases the number of myeloid cells and B cells in the bone marrow immune system [6]. Hematopoietic stem cells in the bone marrow can engulf macrophages in an environment with high fat content. Proinflammatory factors and reactive oxygen species can promote matrix metalloproteinases, tumor necrosis factor (TNF), and interleukin (IL-6) and further increase inflammation, which is mediated by white adipose tissue [22].

Obesity can also lead to insulin resistance and poor glucose tolerance, leading to type 2 diabetes mellitus and ultimately to bone lesions, including decreased cortical density and destruction of bone trabecular structure. The increase in inflammation level is an important factor leading to bone loss [23]. Inflammation decreases bone mass mainly by increasing the number of macrophage colony-stimulating factors and the expression of RANKL in osteocytes. In ovariectomized mice, inhibition of IL-1 or TNF can slow down bone loss [24].

When infectious or noninfectious stimuli enter the body, the body is able to resist the stimuli through soluble factors secreted by immune cells, thereby enhancing the body's defensive response. However, these inflammatory factors, including interferon, IL, and chemokines, can affect the growth and differentiation of osteocytes. These inflammatory factors are also known as inflammatory osteoporosis mediators. The effects of inflammatory factors on osteoblasts and osteoclasts should not be underestimated. First, inflammatory factors can amplify the role of inflammation in progressive transmission and induce other cytokines, noncytokine inflammatory mediators, and proteases. These factors can stimulate osteoblasts and osteoclasts, enhance osteoclast function, and inhibit osteoblast function [25].

Some scholars say that inflammation may be the main cause of bone loss and can cause disability and mortality [26]. In chronic inflammatory diseases, such as periodontitis and rheumatoid osteoarthritis, there is a considerable degree of negative bone mass balance. However, inflammatory bone loss is only a small-scale relief of inflammation, as inflammation usually spread to the entire body, leading to total bone loss. Recent experiments have proven that the treatment of chronic inflammation can prevent the progression of osteoporosis and that tetracyclines are effective in treating bone loss in patients with periodontitis [26]. In cystic fibrosis, persistent infection can lead to total bone loss, and after anti-infective treatment, the patient's bone status is improved. However, many of the causes of inflammation are unclear, so the current treatment of inflammation may be favorable [27]. Although TNF, IL-1, and IL-6 play an important role in the activation and differentiation of osteoclasts, current studies have not fully confirmed the role of proinflammation in the pathogenesis of osteoporosis. Therefore, further research is needed on this area.

Swimming may increase bone density by strengthening muscles. Muscle aging plays an important role in the pathogenesis of osteoporosis. At present, the lack of muscle capacity will lead to osteoporotic fractures. Age, disease, cell aging, decreased physical activity, and decreased sex hormone synthesis are important factors affecting muscle loss [28]. External force acting on the body is mediated by cytoskeleton proteins, which are eventually sensed by the nucleus. The detection of muscle function is very difficult at present. Men and women are almost equally susceptible to muscle loss. However, women experience muscle loss earlier than men; thus, the incidence of fracture is higher in women with rapidly changing hormone levels after menopause, which has great effect on life quality and health [29].

Swimming is the best way to prevent and treat osteoporosis when taking into account side effects, but other methods are still needed to manage and treat osteoporosis. While inhibiting osteoclasts, promoting the differentiation and maturation of osteoblasts is also an effective measure to treat osteoporosis. Currently, effective measures include intermittent use of monoclonal antibodies, parathyroid hormone (PTH), and Dickkopf WNT Signaling Pathway Inhibitor 1 (DKK1). Some researchers used PTH in the experimental model of fracture arthritis, which eventually reversed bone loss and repaired local bone destruction [30]. Therefore, tripamine, a 1-34 amino acid fragment of PTH, has the effect of reversing bone loss. DKK1 and sclerostin inhibit osteoblasts and osteoblasts mainly through the WNT signaling pathway. At present, the humanized monoclonal antibody against DKK1 is in the stage of clinical trial. Sclerostin's monoclonal antibody is currently being tested in clinical trials, and the effect is very obvious in preclinical and clinical trial stages [31].

The strength of this article is its finding that swimming may improve the BMD of participants. BMD plays a decisive role in osteoporosis, leading to an increased risk of osteoporosis. Unlike previous studies, the researchers used swimming as a placebo for other exercises. The findings of this paper may be used as a reference for the effect of swimming on osteoporosis. Of course, more high-quality studies will be needed in the future to further confirm this conclusion.

## 5. Conclusions

Although more clinical randomized controlled trials are needed to study the effect of swimming on BMD in other parts of the human body, we have preliminary evidence to show that swimming may have an effect on the lumbar vertebra density of premenopausal swimmers and that swimming may improve the BMD or the radius in these participants. This may also be a good program for the clinical prevention and treatment of osteoporosis.

## Figures and Tables

**Table 1:** Characteristics of the included studies.

Study year	Journal	Country	Design	Mean age		Simple size		Relevant outcomes
				Swimming	Placebo	Swimming	Placebo	
Andreoli 2012	*European journal of clinical nutrition*	U.K.	Retrospective study	58.4	60.8	12	24	Total body and regional BMD, BMC, fat mass and lean body mass
Czeczuk 2012	*Advances in clinical and experimental medicine*	Poland	Case control study	50.7	52.1	18	18	Body fat, BMC, BMD
Mohr 2015	*European journal of applied physiology*	Germany	Randomized controlled trials	46	45	21	20	BMD, BMC, P1NP, CTx
Greenway 2012	*European journal of applied physiology*	Germany	Case control study	40.4	43.8	43	44	BMD, BMC
Nagata 2002	*Journal of physiological anthropology*	Japan	Retrospective study	59.7	60.9	41	22	BMD, height, body weight, and BMI

DB: double-blind; P1NP: procollagen type 1 amino-terminal propeptide; CTx: carboxy-terminal crosslinking telopeptide of type I collagen; BMD: bone mineral density; BMC: bone mineral content.
